# A Novel Subcluster of Closely Related *Bacillus* Phages with Distinct Tail Fiber/Lysin Gene Combinations

**DOI:** 10.3390/v15112267

**Published:** 2023-11-17

**Authors:** Rachel E. Loney, Véronique A. Delesalle, Brianne E. Chaudry, Megan Czerpak, Alexandra A. Guffey, Leo Goubet-McCall, Michael McCarty, Madison S. Strine, Natalie T. Tanke, Albert C. Vill, Greg P. Krukonis

**Affiliations:** 1 University Program in Genetics and Genomics, School of Medicine, Duke University, Durham, NC 27708, USA; 2 Department of Biology, Gettysburg College, 300 N Washington St., Gettysburg, PA 17325, USA; czerme01@gettysburg.edu (M.C.); mccami02@gettysburg.edu (M.M.); 3 LGC Clinical Diagnostics, 910 Clopper Rd., Gaithersburg, MD 20874, USA; tomkobri12@gmail.com; 4 Janssen Scientific Affairs, LLC. 200 Tournament Dr., Horsham, PA 19044, USA; aagesen22696@gmail.com; 5 Department of Biology, The Pennsylvania State University, 201 Huck Life Sciences Building, University Park, PA 16802, USA; ljg5555@psu.edu; 6 Department of Immunobiology, Yale School of Medicine, 333 Cedar St., New Haven, CT 06510, USA; madison.strine@yale.edu; 7 Department of Cell Biology and Physiology, University of North Carolina, Chapel Hill, NC 27599, USA; ntanke22@live.unc.edu; 8 Department of Ecology and Evolutionary Biology, Yale University, New Haven, CT 06520, USA; albert.vill@yale.edu; 9 Department of Biology, Angelo State University, Cavness Science Building 101, ASU Station #10890, San Angelo, TX 76909, USA; greg.krukonis@angelo.edu

**Keywords:** bacteriophage, *Bacillus subtilis*, comparative genomics, horizontal gene transfer, functional annotation, tail fiber/lysin gene combinations

## Abstract

Bacteriophages (phages) are the most numerous entities on Earth, but we have only scratched the surface of describing phage diversity. We isolated seven *Bacillus subtilis* phages from desert soil in the southwest United States and then sequenced and characterized their genomes. Comparative analyses revealed high nucleotide and amino acid similarity between these seven phages, which constitute a novel subcluster. Interestingly, the tail fiber and lysin genes of these phages seem to come from different origins and carry out slightly different functions. These genes were likely acquired by this subcluster of phages via horizontal gene transfer. In conjunction with host range assays, our data suggest that these phages are adapting to hosts with different cell walls.

## 1. Introduction

The past several decades have seen a renewed interest in bacteriophages (phages) from both ecological and medical perspectives. Phages enact strong selective pressure on host bacterial species [1,2] and thus play a large role in shaping microbial ecology [1,3,4,5]. Phages also play a role in biogeochemical cycling by lysing their hosts and shunting host material back into the pool of organic matter [6] and by altering the gene expression and therefore the metabolism of their hosts [7,8]. Phages are also known to impact human health via their role in shaping the gut microbiome [9,10,11], and phage therapy has become an attractive option as more and more human pathogens acquire antibiotic resistance [12].

However, phages largely remain the “dark matter” of the biosphere [13]. With an estimated 10^31^ bacteriophage particles in the world [14], we have barely scratched the surface of the vast phage diversity that exists around us. Aside from the sheer quantity of phages that have not yet been isolated, within the phages that we have sequenced, there is a plethora of genes with unknown functions [15,16,17]. Isolating and sequencing more phages will allow us to more fully describe phage genetic diversity, and will provide a better framework for understanding how different genes and functions impact phage ecology. Expanding our knowledge of phage genetics will allow for advancements in identifying and utilizing new phage genes that attack bacteria, uncovering how phages can manipulate bacterial gene expression and metabolism, and further understanding the co-evolutionary dynamics between phages and their hosts.

To date, most phage research has focused on Actinophages [17,18,19], phages found in aquatic environments [6], and lab-adapted phages (such as Lambda, T phages, or SPP1) [20]. Our goal has been to document the diversity of phages that are able to lyse “wild” bacterial isolates that have experienced little lab domestication. This will allow for a better picture of phage diversity as it exists in nature. To that end, we have focused on phages that can lyse various strains of *Bacillus subtilis* [20,21,22]. *B. subtilis* is a highly diverse Gram-positive bacterium typically found in soil [23], and its ability to form endospores facilitates its successful survival in arid desert soil [24,25,26]. In addition to being a model organism, *B. subtilis* is of interest for many reasons including its capacity for promoting plant growth [27] and its role as a symbiont in the digestive tracts of animal hosts [28,29].

Here, we describe seven phages that we isolated from desert soil from the southwest United States and which constitute a novel subcluster sharing little genetic similarity with previously described phages. As seen in many other phage genomes [16,17,30,31,32,33,34], our phages show evidence of having acquired many genes through horizontal gene transfer (HGT). While many of these are small genes with unknown functions, there is some indication that these phages are adapting to differences in host cell walls.

## 2. Materials and Methods

Soil was collected from various locations in the southwest United States (Table 1). At each collection site, the GPS coordinates were recorded (Table 1), and soil was collected into a sterile 50 mL conical tube. To isolate phages from each sample, 1 g of soil was added to 20 mL of LB broth containing 10 mM CaCl_2_, 10 mM MgCl_2_, and 1 mM MnCl_2_ (LB2), incubated for 4 h at 37 °C shaking at 250 rpm, and stored at 4 °C after filtering (0.22 μm). A sample of filtrates was then plated on *B. subtilis* strains: either on wild strains T89-06 (also called S89-6 or T89-6), T89-20 (also called S89-20), or TT123, all isolated by Istock and colleagues [35,36] or on the lab-adapted strain 168 [23] which was acquired from the Bacillus Genetic Stock Center. Each of these *B. subtilis* strains are genetically distinct from each other and are kept as spore stocks in our lab (described in [20]). Phages were isolated by single-plaque purifying three times on lawns of the isolation host. Phages were successfully recovered from 1 g of soil from each collection site. Two phages were isolated from 1 g of soil taken from Death Valley, while only one phage was isolated from the other soil samples. High-titer lysates (HTL) were prepared by flooding multiple webby plates with LB broth and filtering the lysate (0.22 μm). DNA was extracted from phage 268TH004 and the phages from Big Bend following the phagesdb phenol–chloroform extraction procedure [37] and from the other phages with the Promega Wizard DNA Clean-Up System.

Phage DNA was sent to North Carolina State University’s Genomic Science Laboratory where libraries were prepared with the Illumina Truseq Nano DNA library prep kit and sequenced on Illumina MiSeq (v3 150 SE flow cell). Upon receiving sequencing data, reads were aligned and assembled using GS de novo assembler v2.9 [38], and quality was verified with Consed v29 [39]. Each genome was assembled as a single contig with >1000× coverage. Genome ends were determined with PhageTerm [40].

Finished sequences were annotated within DNAMaster v5.22.22 [41]. Putative genes were called with Glimmer v3.0 and Genemark v2.5 [42,43]. Predicted protein functions were called with BLAST v2.12 (if E-value < 10^−5^) [44] and HHPred (if probability > 85%, coverage > 50%, and E-values < 10^−5^) [45]. The presence of tRNA genes was confirmed with Aragorn [46]. Default settings were used in all programs, and all genome annotations have been submitted to NCBI (accession numbers can be found in Table 1). For select genes of interest, predicted domain functions were called with HHPred and with NCBI’s Conserved Domain Database (CDD) using RPS-BLAST [47] via Phamerator [48].

A Phamerator database was constructed with 312 *Bacillus* phages with complete genomes in NCBI as of 1 May 2022 (Bacillus v5—https://phamerator.org) [48]. This database groups gene products together into protein families with related sequences (“phams”) using the PhamMMseqs pipeline, and phages that share at least 35% of their phams are described as belonging to the same cluster [49]. Genome maps were created with Phamerator to illustrate the relationships between phage genomes using both amino acid and nucleotide similarity. Additional genomic comparisons were made by (1) calculating the average amino acid identity (AAI) and average nucleotide identity (ANI) using enve-omics lab algorithms [50], (2) performing BLASTn (nr/nt database) and BLASTp (nr database) searches (performed July 2023) either against the complete database or against the database for tailed phages (taxid: 10699, 10662, and 10744), or (3) aligning amino acid sequences of homologous genes using MUSCLE [51] in MEGAX (default parameters) [52]. Alignment figures were made with ESPript3.0 [53].

Representative brightfield TEM images were taken of phage 019DV002. First, 1 mL of 019DV002 HTL was centrifuged at 10,000× *g* for 1 h at 4 °C. TEM grids (Ted Pella, Inc., Redding, CA, USA, Prod. #01822) were treated with the concentrated phage particles, washed briefly with ddH_2_O, and then stained with 2% phosphotungstic acid. Images were taken at 130,000× magnification using a Philips CM1000 TEM.

We tested the phages’ ability to lyse various *B. subtilis* strains using a microtiter plate-based assay [54] with an MOI of 0.1 using 9.1 × 10^7^ PFU/mL and 9.1 × 10^8^ CFU/mL. Phage and bacteria were plated in 220 μL of LB2 broth in a 96-well plate, and each combination of phage and bacteria was replicated five times. Wells with bacteria but without phage served as controls. Phages were tested against seven strains: two lab strains 168 (BGSCID 1A1) and W23SR (BGSCID 2A3) [55] and five genetically distinct wild strains in our lab collection originally from [35,36]. Plates were incubated for 20 h at 37 °C with auto-mixing for 10 s every 5 min, and absorbance at 600 nm was measured every 30 min. For a conservative estimate, a phage was said to impact bacterial growth if at least 4 of the 5 replicates showed lower absorbance readings than the control bacteria. To complement the plate assay data, spot testing phages on each host was also performed by spotting 10^4^ PFU on a lawn of each host and confirming presence or absence of plaques.

## 3. Results

### 3.1. Basic Genomic Data

**Figure 1:**
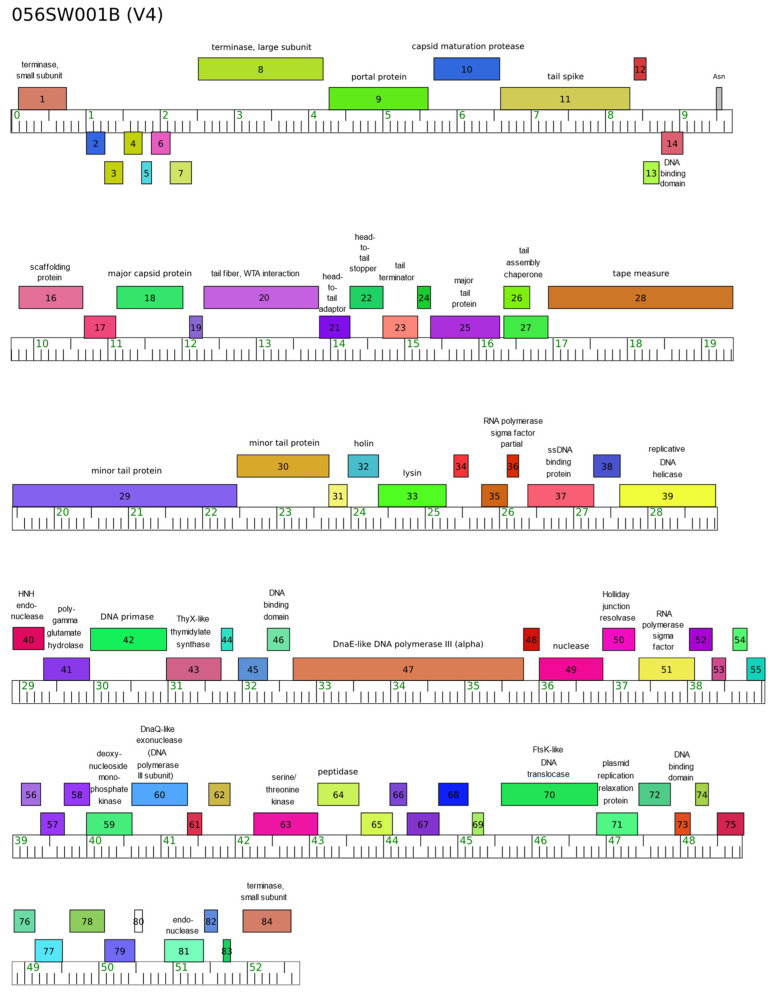
Genome map of 056SW001B, chosen because it shares phams with some but not all of the other phages in this subcluster. The ruler shows genome length (in kilobases) with forward and reverse genes shown above and below the ruler, respectively. Functions or putative functions are listed above genes. Due to direct terminal repeats, gene 84 is a duplication of gene 1 (terminase small subunit). Map was created using Phamerator [48].

**Figure 2:**
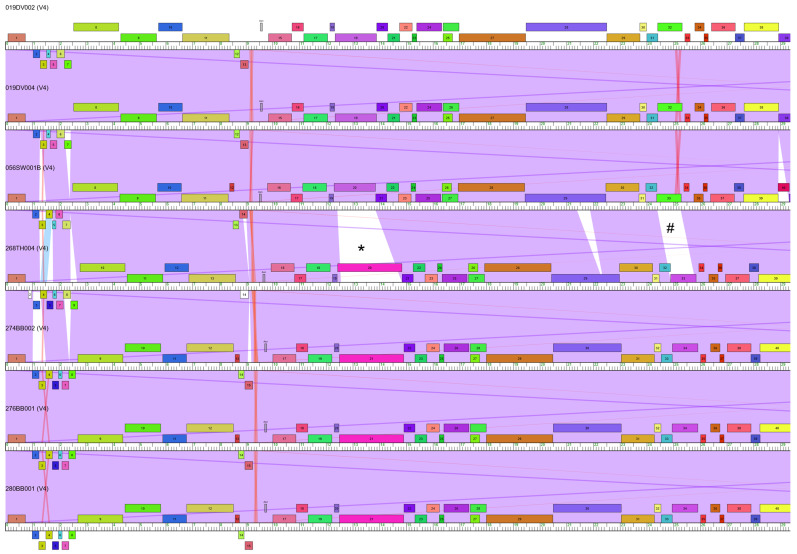

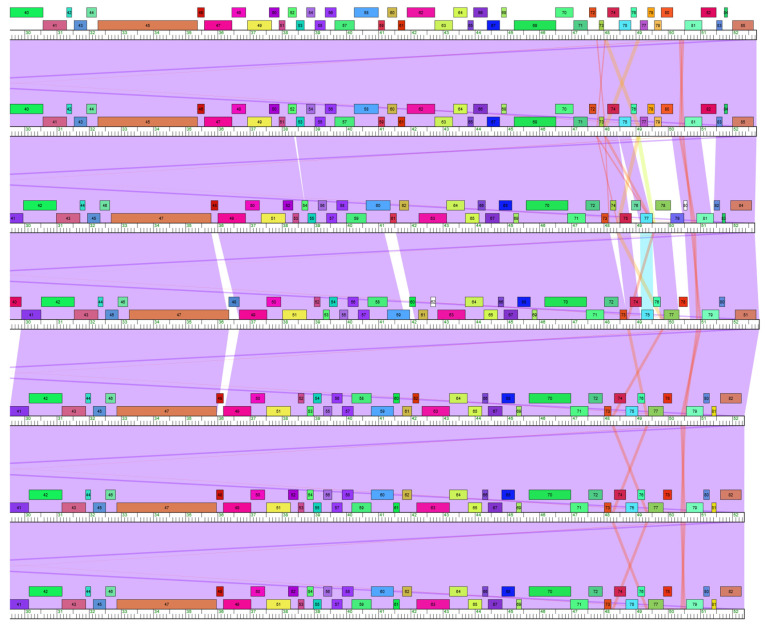
Whole genome maps of the V4 phages, created with Phamerator [48]. Nucleotide sequence similarity between adjacent genomes is indicated by shading from lavender (most similar) to red (least similar). A lack of shading shows that there is no similarity based on local alignment E score greater than 10^−4^. * marks the WTA-tail fiber/peptidase phams, # marks the lysin/endolysin phams. A total of 96 phams were identified among these seven phages.

**Figure 3:**
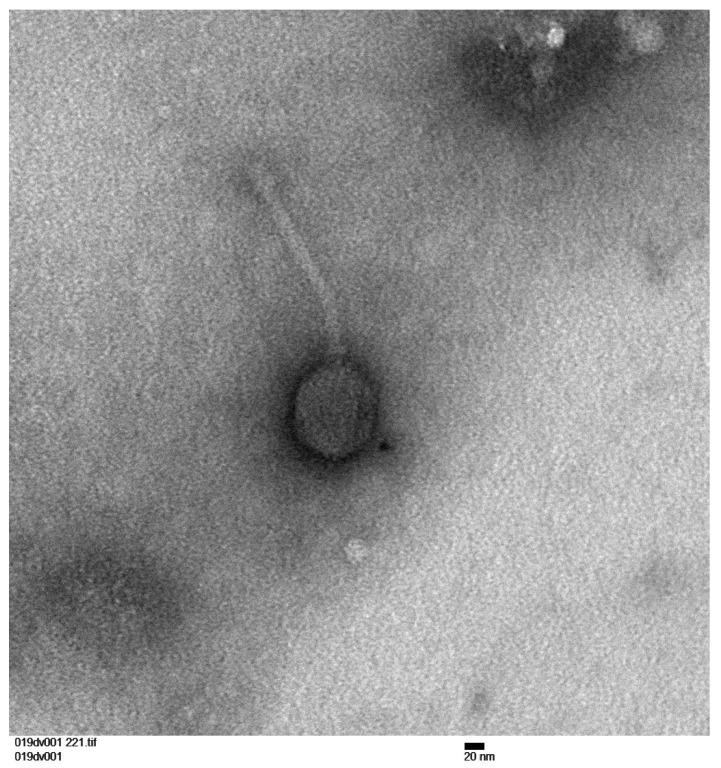
Transmission electron microscopy image of phage 019DV002. Image taken at 130,000x using a Phillips CM1000 TEM.

Seven *B. subtilis* phages were isolated from soil collected in the southwest United States (Table 1). 280BB001 was isolated on the lab strain *B. subtilis* 168, and the other six phages were isolated on wild strains of *B. subtilis* (Table 1). These phages have double-stranded DNA genomes with 839 bp direct terminal repeats and a GC content (42.4–42.6%) quite close to *B. subtilis*’s GC content of 43.5% [56] (Table 1). The genomes range from 52,370 to 52,834 bp in length and contain 80–84 putative protein-coding genes (Table 1, Figure 1 and Figure 2). All seven phages have the same Asn-GTT tRNA gene, located just before the scaffolding gene (Figure 1), and no other tRNAs. Each phage makes clear plaques on *B. subtilis* lawns, indicating that they are lytic phages. This conclusion is supported by the lack of integrase protein-coding genes in their genome annotations. Both genome organization and electron microscopy (Figure 3) support that these phages are siphoviruses [16].

These phages show high average nucleotide and amino acid identity (ANI and AAI, respectively) when compared to each other, ranging from 95.4% (ANI) or 91.7% (AAI) to 100% (Table 2). Based on their high similarity to each other and using the Phamerator clustering program, these seven phages have been classified as belonging to their own subcluster, which is designated as V4. The phages isolated from the same locations are quite similar to each other with 100% ANI between the Death Valley (DV) phages and ≥99.9% ANI between the Big Bend (BB) phages. The DV phages differ by only two non-synonymous SNPs in one gene (gene 19, tail fiber). 274BB002 differs from the other BB phages with two 1 bp intergenic insertions (a G at position 838 and a C at position 51,533, both present in 274BB002 only). 280BB001 differs from the other BB phages with one 1 bp intergenic indel (8575 in 280BB001: 16 vs. 15 T). Finally, 276BB001 and 280BB001 also differ by one non-synonymous SNP (base 33,057) in gene 47 (DnaE-like DNA polymerase III alpha). Although the DV phages were isolated from the same gram of soil, the BB phages were isolated from soil samples between 14 and 35 km apart from each other. Whole genome BLASTn searches show that the next best hit to these seven phages, besides each other, is *Bacillus pseudalcaliphilus* phage vB_BpsS-36 from subcluster V2 (coverage = 29%, identity = 69%).

Among the V4 phages, we have identified 96 phams (“phamilies” of related protein sequences [18,48]). Of those phams, 64 are shared by all seven phages, with 19 phams that are unique to the V4 subcluster (based on the 312 phages in the *Bacillus* Phamerator database). Only two of the unique phams shared by all V4 phages have functional annotations: a tail assembly chaperone and a partial RNA polymerase sigma factor. Of the remaining 32 phams shared by six or fewer V4 phages, 15 are exclusive to the V4 subcluster. These 15 phams include 4 orphams (phams with a single phage member): 3 from 268TH004, and 1 from 056SW001B. None of these orphams have functional annotations.

### 3.2. Horizontal Gene Transfer in Subcluster V4 Evolution

Despite the high pairwise AAIs across phages in this subcluster, a third of the phams that we identified (32 out of 96) are not shared by all V4 phages. We see evidence of HGT in how these 32 phams are distributed across the V4 phage genomes. Most of these phams are fairly small, which is unsurprising given that smaller genes may be more readily exchanged [17]. These phams tend to have no known function, and tend to be inserted between genes that are conserved across the V4 phages (Figure 2).

However, there are two instances in the V4 genomes where a large gene with a functional annotation has been fully replaced by a different gene. First, where the DV (gene 19) and Saguaro West (SW) (gene 20) phages have a tail fiber gene, the Tumamoc Hill (TH) (gene 20) and BB (gene 21) phages have a gene that has been annotated as a peptidase but likely functions as a tail fiber (Figure 2, marked with *). For clarity, the DV and SW gene will be referred to as the “WTA-tail fiber” (wall teichoic acid–tail fiber), and the TH and BB gene will be referred to as the “peptidase.” The second instance involves the same phages with different lysin genes in the DV (gene 32) and SW (gene 33) phages versus the TH (gene 33) and BB (gene 34) phages (where the lysin gene product is more specifically called a LysM-like endolysin) (Figure 2, marked with #). For clarity, the DV and SW gene will be referred to as the “lysin” and the TH and BB gene will be referred to as the “endolysin.” Outside of the V4 phages, there are no phages who share these same pairs of phams in the current *Bacillus* Phamerator database; the WTA–tail fiber is only present in V4 phages, and the endolysin/peptidase combination is only seen in V4 phages.

#### 3.2.1. Tail Fiber

The WTA-tail fiber and peptidase genes likely carry out similar functions, but it appears that they come from different origins. The WTA-tail fiber gene has sequence similarity to the receptor binding protein (RBP) of *Staphylococcus aureus* siphophage ϕ11 [57]. The ϕ11 RBP assembles into a homotrimer that is required for ϕ11 adsorption onto wall teichoic acids (WTA) [57], which are a key component of Gram-positive bacterial cell walls and known to be receptors for phage adsorption [58,59,60,61]. Interestingly, this is the gene where the two SNPs in the DV phages are found (at positions 12,866 and 13,757). In the TH and BB phages, the peptidase gene contains a C-terminal G2 peptidase domain that has been identified as the tail spike protein in a number of bacteriophages [62], supporting the assumption that the peptidase likely performs the same function as the DV and SW phage tail fiber.

**Figure 4:**
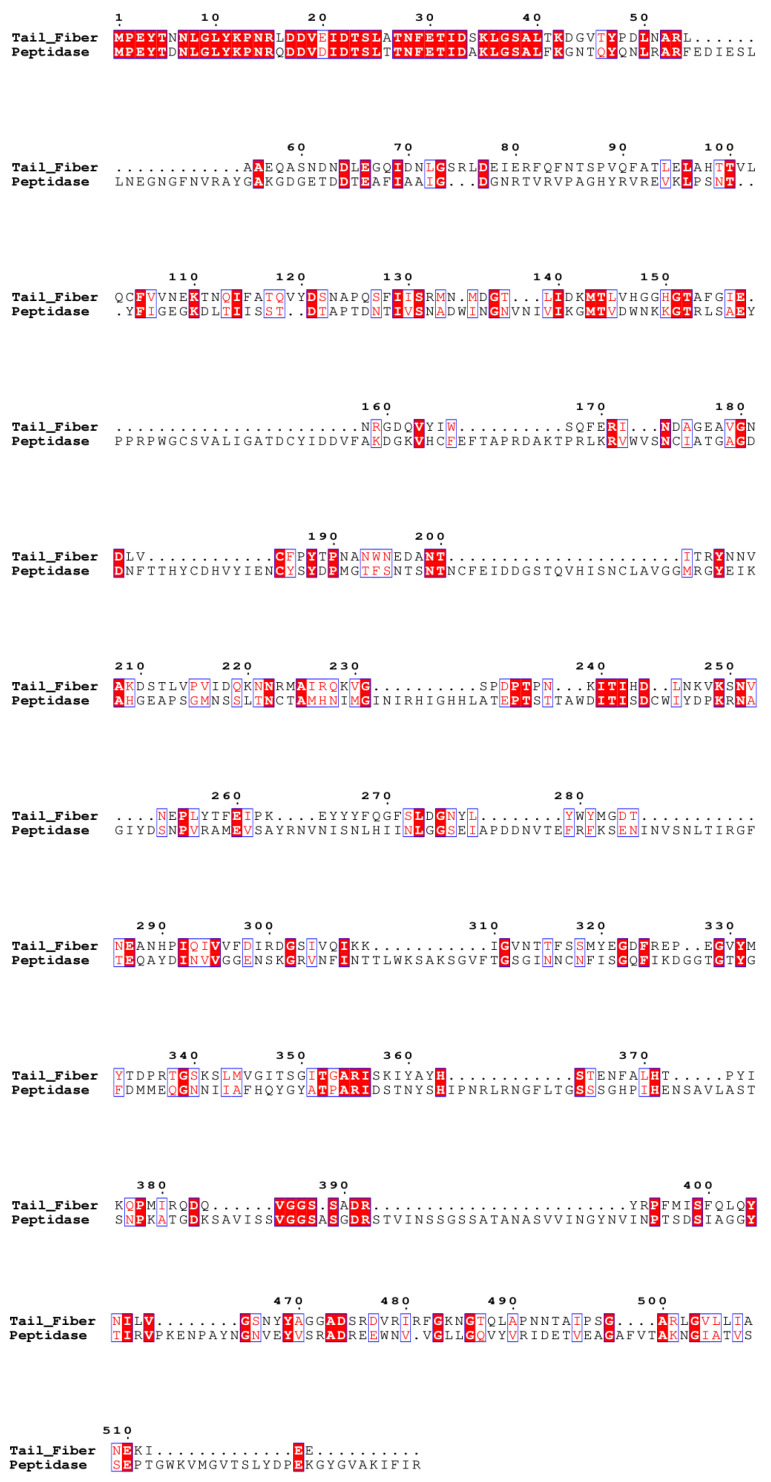
MUSCLE alignment of 056SW001B gene 20 (Tail_Fiber) and 268TH004 gene 20 (Peptidase), made with [52,53].

The WTA-tail fiber pham is only present in the V4 subcluster, while the peptidase pham is found in 15 other *Bacillus* phages: subclusters AA, B1, D2, and U. Subcluster AA is composed of two phages that our lab isolated from Tumamoc Hill, which is the isolation site for 268TH004 [22]. In the V4 subcluster, the WTA-tail fiber/peptidase gene is located between the major capsid protein (MCP) and the head-to-tail adaptor (HTA). However, the other V phages do not share gene synteny here, and they either contain smaller genes that lack a structural function (subclusters V1, V2, V5) or have no gene separating the MCP and HTA (subcluster V3). Additionally, the next best BLASTp hits to the WTA-tail fiber are all to teichoic acid biosynthesis proteins in various *Bacillus* species (identity ≤ 43%) rather than to other phages. Similarly, the next best BLASTp hits to the peptidase are to peptidase G2 autoproteolytic cleavage domain-containing proteins in various *Bacillaeceae* species (identity ≤ 68%) rather than other phages. The break in gene synteny among V phages, the sharing of the peptidase pham with phages in a different cluster that are geographically near 268TH004, and the BLASTp results suggest that these genes were acquired by the V4 phages through HGT. We note, however, that the first ~50 amino acid residues are highly conserved between the WTA-tail fiber and peptidase (Figure 4). This region is upstream of the first functional domain of either protein. It is possible that this region was present in the V4 common ancestor and was not recombined with the rest of the gene, although there is still no notable homology between this region and the other V subclusters.

#### 3.2.2. Lysin

As is typical for Gram-positive bacteriophages [63], the V4 lysin proteins each have an N-terminal catalytic domain and a C-terminal domain that binds to the bacterial cell wall. The DV and SW lysin’s N-terminal domain is a muramidase in the GH25 family and is predicted to cleave the 1,4-beta-linkages between the N-acetylmuramic acid and N-acetyl-D-glucosamine residues, which construct the peptidoglycan backbone [64]. The TH and BB endolysin’s N-terminal domain is an N-acetylmuramoyl-L-alanine amidase predicted to break the amide bond between N-acetylmuramoyl and L-amino acids, which is a key component of the cross-linked peptide chains of peptidoglycan [64]. Both genes have a C-terminal LysM domain which is predicted to bind to peptidoglycan.

**Figure 5:**
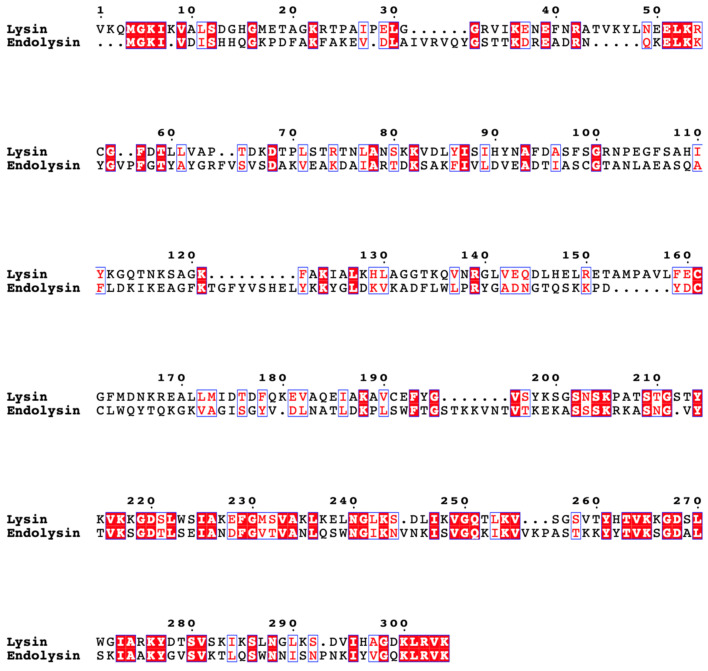
MUSCLE alignment of 056SW001B gene 33 (Lysin) and 268TH004 gene 33 (Endolysin), made with [52,53].

MUSCLE alignment of the lysin and endolysin genes reveals low homology between them in the N-terminal region (Figure 5). The N-terminal domains of the lysin and endolysin (identified by the CDD as residues 10-183 in the alignment) share only 17% identity in their amino acid sequences (30 residues out of 173). The C-terminal domains of these genes (identified by the CDD as residues 261-303) share 44% identity (19 residues out of 43). Phage lysins can retain their C-terminal domains while swapping out new N-terminal domains in order to cleave different peptidoglycan bonds [19,63], and we predict that the differences between the V4 lysin and endolysin are a product of one of these recombination events.

Overall, 13 other *Bacillus* phages outside of the V4 subcluster share the endolysin pham (across subclusters A5, J, X, Y2), and 33 other *Bacillus* phages (subclusters B2, B3, B4, E, G, V1, Y1, and 6 singletons which do not have cluster assignments) and 1 *Geobacillus* phage (subcluster V3, phage GBK1) share the lysin pham. There is gene synteny between the cluster V phages, as the location of the lysin is conserved between the V1 and V4 phages. Interestingly, while the endolysin has multiple BLASTp hits to other *Bacillus* phages, the lysin’s best BLASTp hits (after the V4 phages) are to various *Bacillaceae* species (coverage ≤ 83%, identity ≤ 73%) rather than to other phages. The differences in N-terminal domain function, low homology between the N-terminal domains, and BLASTp results suggest that the V4 phages acquired at least the lysin/endolysin N-terminal domains, if not the whole gene, from HGT.

#### 3.2.3. Other Genes

In addition to the above two pairs of genes, there is an indel in the minor tail protein that further segregates the V4 phages. All of the V4 phages share the same pham for the minor tail protein (gene 29 in 056SW001B), but this gene is 480 bp longer in the DV and SW phages than it is in the TH and BB phages (Figure 2). The non-conserved indel region contains a domain in the carbohydrate-binding module (CBM) 4_9 family.

The WTA-tail fiber/peptidase and lysin/endolysin genes account for 4 of the 32 phams that are only present in some, but not all, of the V4 phages. Of the remaining 28 phams, only 2 have functional annotations. First, all of the V4 phages except for 268TH004 share a gene that codes for a DNA-binding domain (gene 14 in 056SW001B) nestled within the structural cassette between the tail spike and the scaffolding protein (Figure 1). Given the presence of other genes coding for DNA binding domains in the genome, including one shared by all V4 phages (gene 46 in 056SW001B), it is unclear how the lack of this pham may impact 268TH004’s life cycle or fitness. Second, the BB phages all lack a gene coding for an HNH endonuclease present in the other V4 phages. This pham’s location is not perfectly conserved across the other four phages. It is located in the middle of the replication cassette in the SW and TH phages (gene 40), but in the DV phages it is located at the end of the genome among genes with no known function (gene 82). Homing endonucleases are often found as free-standing genes inserted amongst areas of the genome that are otherwise conserved between related phages [65], so their presence in only some of the V4 phages is unsurprising.

The remaining 26 phams that are only present in some of the V4 phages have no known function, making it difficult to draw any conclusions about the fitness implications of their presence or absence. We do note that while all V4 phages have the same complement of genes involved in DNA metabolism (see Figure 1 for functions), several of these genes of unknown function are scattered in the replication cassette. First, after the DnaE-like DNA polymerase III (alpha) (gene 47 in 056SW001B), each V4 phage has a small gene of unknown function, but 268TH004’s gene is in a different pham than the rest. The pham found in 268TH004 is also found in a cluster W3 myovirus, which was also isolated from soil in the southwest United States by our lab (phage 035JT004 from Joshua Tree National Park, CA, USA). Second, after the DnaQ-like exonuclease (DNA polymerase III subunit, gene 60 in 056SW001B), the DV and SW phages share one pham of unknown function, while the TH and BB phages share a different pham of unknown function (Figure 2). Both of these phams are only present in the V4 phages.

### 3.3. Host Range

We tested the host range of our phages against seven *B. subtilis* strains, including five wild strains kept by the lab and the domesticated strains 168 and W23. We chose to assess host range using a microtiter plate-based assay, where a depression or delay in the bacterial growth curve indicates that phage are killing the host. Laboratory methods of determining host range can be inconsistent or only show an incomplete measure of a phage’s infective ability [4,66]. To counter this, we opted for a conservative measure of host range where we judged a phage as able to infect a particular host if phage presence depressed bacterial population growth compared to the no-phage controls in at least four out of five replicates. These observations were also complemented by spot test assays on bacterial lawns.

All of the V4 phages except for 268TH004 and 274BB002 were able to infect 168 (Table 3). All V4 phages were able to lyse W23 with the caveat that 276BB001 was able to plaque on a lawn of W23 but unable to substantially depress the bacterial population in liquid media (Table 3). For four of the wild strains, the V4 phages largely had the same infective capability. However, only the DV and SW phages were able to lyse the fifth wild strain, T89-30 (Table 3). This split follows the same division in the V4 phages as the long/short minor tail protein, WTA-tail fiber/peptidase, and lysin/endolysin phams described above.

## 4. Discussion

Here, we described seven recently isolated *Bacillus subtilis* phages from the southwest United States. Given that relatively few *Bacillus* phages have been described [20], it is not surprising that isolating these phages allowed us to describe a novel subcluster. More surprisingly, we were able to identify highly related phages belonging to the same subcluster across a broad geographic range, as some of our collection sites were ~1500 km apart. Microbial dispersal is of great ecological significance [67], and the isolation of three nearly identical phages 14–35 km apart underscores the dispersal capacity of phages.

Despite the high ANI/AAI among these phages, only two thirds of the phams that we identified are conserved across all seven phages. The non-conserved phams tend to be small and have no known function, which is a common finding in other phages as well [15,17]. Some studies suggest that the gene products of small, non-conserved phage genes may act as a “molecular splint” and bind to host proteins to inhibit or modify their function, as has been described with some small T4 genes [15], and that may well be the case with some of these V4 phams. Further advances in protein modeling or the crystallization of these small proteins may give additional insight into the putative functions of these genes. Comparative analyses may also identify gene functions as more phage and host bacteria genomes are sequenced and explored.

Our data provide additional evidence that phage genomes are mosaic with HGT as a key player in phage genome evolution [16,17,30,31,32,33,34]. The V4 phages largely differ by the presence/absence of small genes or regions, which is a common finding in phage genomes. It is possible that these regions were acquired or shared by the V4 phages through HGT, although further computational studies would be required to determine their origin. We also observed two sets of functional genes that were acquired either entirely or modularly through HGT, which were notable given their large size.

The division in the V4 phages between the WTA-tail fiber/peptidase and lysin/endolysin phams, as well as the indel of a functional domain in the minor tail protein, all suggest that these phages are adapting to differences in the host cell walls that they encounter. The differences in the minor tail protein and the tail fibers suggest that these phages may bind to different cell wall substrates, although our comparative analyses cannot be conclusive in this regard. The division between the lysin and endolysin genes suggests that the phages use different strategies for lysing host cell walls. We do note that the enzymatic domains contained in these phams are very common in phages [19] and break very conserved and common bonds in peptidoglycan [68]. Nevertheless, the fact that phages with the long minor tail, WTA-tail fiber, and lysin gene are unanimously able to infect host T89-30 while the other V4 phages cannot indicates that these genes do play a role in determining host range. Perhaps these genetic differences speak to the changing availability of certain hosts between California, Arizona, and Texas, or a shift in the structure of host cell walls due to differing abiotic conditions across that geographical range. We would need a better understanding of how *B. subtilis* cell walls vary by strain and environment to fully understand how the different V4 tail fiber and lysin gene products impact V4 phage ecology. Additionally, further work to characterize host genomes, such as by determining the presence/absence of certain phage receptors or differences in cell wall composition (e.g., type of cell wall teichoic acids), will enhance our framework for understanding V4 phage ecology and evolution.

Phage diversity remains the “dark matter” of the biosphere with relatively very few sequenced phage genomes compared to the number of sequenced bacterial genomes [13]. As the phage community continues to identify and describe new phage isolates, we will undoubtedly characterize additional novel phage clusters and gain a fuller picture of bacterial and viral ecology [14]. Integrating host range data with genomic data may help us to map additional gene functions. Future studies comparing data generated by different host range methods would aid us in linking genomic features to functional host range effects especially when combined with studies determining at what stage of the life cycle (e.g., restriction, adaptive immunity, abortive infection) some phage/strain combinations are prevented from productive infection. Once a sufficient number of *Bacillus* strains and associated phages have been isolated and annotated, future work integrating what we know of phage and host bacteria diversity and ecology into a comprehensive review will give us much-needed insights into how phages adapt to their hosts in a natural environment.

## Figures and Tables

**Table 1:** Basic genomic characteristics of seven novel *B. subtilis* phages. ORF = Open Reading Frame signifying either known or putative gene; * = includes one Asn-GTT tRNA gene; NP = National Park. Genome size includes 839bp direct terminal repeats. Phages 019DV002 and 019DV004 were isolated from the same gram of soil.

Phage	Isolation Strain	Genome Size (bp)	% GC	No. of ORFs	Collecting Site and Date	GPS Coordinates	Accession
019DV002	T89-06	52,749	42.6	84 *	Death Valley NP, Ca, May 2014	36°27′51.4″ N, 117°14′08.6″ W	MN176220
019DV004	T89-06	52,749	42.6	84 *			MN176221
056SW001B	T89-20	52,692	42.6	83 *	Saguaro West NP, AZ, May 2014	32°16′12.7″ N, 111°12′20.6″ W	MN176230
268TH004	TT123	52,834	42.4	80 *	Tumamoc Hill, Tucson, AZ May 2016	32°13′04.9″ N, 111°00′12.9″ W	MW394467.1
274BB002	TT123	52,372	42.5	81 *	Big Bend NP, TX, June 2016	29°15′54.6″ N, 103°07′24.5″ W	MZ501264
276BB001	TT123	52,370	42.5	81 *		29°18′16.6″ N, 103°15′52.9″ W	MN176231
280BB001	168	52,371	42.5	81 *		29°18′29.0″ N, 103°28′42.6″ W	MN176232

**Table 2:** Average nucleotide identity (ANI, above the diagonal) and average amino acid identity (AAI, below the diagonal) for the V4 phages. Data presented as percentage and calculated using algorithms from [50].

	019DV002	019DV004	056SW001B	268TH004	274BB002	276BB001	280BB001
019DV002	---	100	96.7	95.4	96.2	96.2	96.2
019DV004	100	---	96.7	95.4	96.2	96.2	96.2
056SW001B	94.6	94.8	---	95.4	96.4	96.4	96.4
268TH004	91.2	92.3	93.2	---	98.1	98.1	98.1
274BB002	92.3	92.7	92.4	95.5	---	100	99.9
276BB001	92.4	92.8	92.4	95.3	100	100	100
280BB001	92.4	92.8	92.4	94.9	100	100	---

**Table 3:** Host range data for the V4 phages on *B. subtilis* hosts. Y = the phage can infect the host, N = the phage cannot infect the host. Y* = the effect of the phage on bacteria growth was small but noticeable. N** = spot testing data suggest the phage can lyse the host on solid media, but this was undetectable in liquid assay.

	Bacterial Strain						
	Domesticated		Wild				
Phage	168	W23	TG115	T89-05	T89-06	T89-20	T89-30
019DV002	Y*	Y	N	Y	Y	Y	Y
019DV004	Y*	Y	N	Y	Y	Y	N**
056SW001B	Y	Y	N	Y	Y	Y	Y
268TH004	N	Y	N	Y	Y	Y	N
274BB002	N	Y	N	Y	Y	Y	N
276BB001	Y	N**	N	Y	N**	Y	N
280BB001	Y	Y	N	Y	Y	Y	N

## Data Availability

All genome sequences have been deposited in the GenBank database of NCBI, and accession numbers are listed in Table 1.
